# Synthesis of Poly(2-Acrylamido-2-Methylpropane Sulfonic Acid) and its Block Copolymers with Methyl Methacrylate and 2-Hydroxyethyl Methacrylate by Quasiliving Radical Polymerization Catalyzed by a Cyclometalated Ruthenium(II) Complex

**DOI:** 10.3390/polym12081663

**Published:** 2020-07-27

**Authors:** Vanessa Martínez-Cornejo, Joaquin Velázquez-Roblero, Veronica Rosiles-González, Monica Correa-Duran, Alejandro Avila-Ortega, Emanuel Hernández-Núñez, Ronan Le Lagadec, Maria Ortencia González-Díaz

**Affiliations:** 1 Unidad de Materiales, Centro de Investigación Científica de Yucatán, A.C., Calle 43 No. 130, Chuburná de Hidalgo, C.P. 97205 Mérida, Yucatán, Mexico; vmartinezcornejo10@gmail.com (V.M.-C.); joaquinwr96@gmail.com (J.V.-R.); veronica.rosiles@estudiantes.cicy.mx (V.R.-G.); monica.correa@estudiantes.cicy.mx (M.C.-D.); 2 Facultad de Ingeniería Química, Universidad Autónoma de Yucatán, Periférico Norte Km. 33.5, Chuburná de Hidalgo Inn, C.P. 97203 Mérida, Yucatán, Mexico; alejandro.avila@correo.uady.mx; 3 CONACYT, Departamento de Recursos del Mar, Centro de Investigación y de Estudios Avanzados del IPN, 97310 Unidad Mérida, Yucatán, Mexico; emanuel.hernandez@cinvestav.mx; 4 Instituto de Química, Universidad Nacional Autónoma de México, Circuito Exterior s/n, Ciudad Universitaria, 04510 Ciudad de México, Mexico; 5 CONACYT–Centro de Investigación Científica de Yucatán, A.C., Calle 43 No. 130, Chuburná de Hidalgo, 97205 Mérida, Yucatán, Mexico

**Keywords:** poly(2-acryloamido-2-methylpropane sulfonic acid), cyclometalated ruthenium(II) complex, radical polymerization, block copolymer, membranes

## Abstract

The first example of quasiliving radical polymerization and copolymerization of 2-acrylamido-2-methylpropane sulfonic acid (AMPS) without previous protection of its strong acid groups catalyzed by [Ru(*o*-C_6_H_4_-2-py)(phen)(MeCN)_2_]PF_6_ complex is reported. Nuclear magnetic resonance (RMN) and gel permeation chromatography (GPC) confirmed the diblock structure of the sulfonated copolymers. The poly(2-acryloamido-2-methylpropanesulfonic acid)-*b*-poly(methyl methacrylate) (PAMPS-*b*-PMMA) and poly(2-acryloamido-2-methylpropanesulfonic acid)-*b*-poly(2-hydroxyethylmethacrylate) (PAMPS-*b*-PHEMA) copolymers obtained are highly soluble in organic solvents and present good film-forming ability. The ion exchange capacity (IEC) of the copolymer membranes is reported. PAMPS-*b*-PHEMA presents the highest IEC value (3.35 mmol H^+^/g), but previous crosslinking of the membrane was necessary to prevent it from dissolving in aqueous solution. PAMPS-*b*-PMMA exhibited IEC values in the range of 0.58–1.21 mmol H^+^/g and it was soluble in methanol and dichloromethane and insoluble in water. These results are well correlated with both the increase in molar composition of PAMPS and the second block included in the copolymer. Thus, the proper combination of PAMPS block copolymer with hydrophilic or hydrophobic monomers will allow fine-tuning of the physical properties of the materials and may lead to many potential applications, such as polyelectrolyte membrane fuel cells or catalytic membranes for biodiesel production.

## 1. Introduction

Poly(2-acrylamido-2-methyl-1-propanesulfonic acid) (PAMPS) displays interesting properties that may lead to many potential applications. Such properties arise from the strongly ionizable sulfonate groups in its chemical structure, its pH-responsiveness and its swelling behavior [1,2,3] AMPS-containing polymers have been successfully applied in polyelectrolyte membrane fuel cells [1], as catalytic membranes for biodiesel production [4] and in medical applications due to their low toxicity, hydrolytic stability and antimicrobial activity against microorganisms [5,6]. Moreover, they are used in a wide range of industrial products such as cosmetics, coatings and adhesives, amongst others [7]. Homo- and copolymers of this monomer have been synthesized straightforwardly by free radical polymerization [8,9,10]. However, it is well known that it is nearly impossible to obtain predetermined molecular weights through the conventional radical mechanism due to chain termination reactions [11]. On the other hand, atom transfer radical polymerization (ATRP) has emerged as a robust technique for synthesizing well-defined polymers with predetermined molecular weights, low dispersity and with different compositions, topologies and functionalities [12,13]. A literature review indicates that in the polymerization of PAMPS by the ATRP technique with copper catalysts, neutralization of the acid groups is required to prevent protonation of the amino-based ligands, which can lead to the decomposition of the copper/ligand catalytic system and to avoid subsequent side reactions [14,15,16,17,18,19]. In many cases, the characteristics of a quasiliving ATRP have not been observed, despite the use of AMPS in its salt form. For example, the synthesis of PAMPS by ATRP in DMF:water solvent (50:50 *v*/*v*) at 20 °C with CuCl and different ligands, such as 2,2′-bipyridine (bpy), *N*,*N*,*N*′,*N*′′,*N*′′-pentamethyldiethylenetriamine (PMDETA) and 1,1,4,7,10,10-hexamethyltriethylenetetramine (HMTETA), presents a nonlinear first-order kinetic plot and experimental molecular weight (M_n,GPC_) higher than its theoretical values, which are indicative of a high concentration of radicals and extensive termination in the initial stage [14]. In that work, for the ATRP synthesis of PAMPS, high catalyst loadings were necessary to reduce its deactivation (CuCl/tris[2-(dimethylamino)ethyl]amine ligand with the addition of equimolar amounts of CuCl_2_ with respect to CuCl) [14,15]. A. Tolstov et al. reported the ATRP of AMPS in aqueous conditions with activators generated by electron transfer (AGET) of sodium 2-acryloamido-2-methyl-*N*-propane sulfonate and its copolymerization with 2-hydroxyethyl acrylate (HEA), using CuBr_2_/HMTETA and L-ascorbic acid as a reducing agent. However, the resulting copolymers presented higher M_n,GPC_ and dispersity (Ð = M_w_/M_n_) than the expected values [16]. On the other hand, the ATRP synthesis of PAMPS was reported with in situ neutralization of AMPS with tri(*n*-butyl)amine (TBA) at 60 °C in dimethylformamide (DMF) with CuCl/bpy catalyst [20]. The authors reported very low conversions (less than 15%), albeit the use of ascorbic acid as a reducing agent improved the reaction rate.

Ruthenium catalysts in ATRP have been described as more tolerant to several functional groups with good solubility in protic and aprotic solvents, which permit quasiliving polymerization in polar media [21,22,23]. In particular, series of cationic 18-electron coordinatively saturated cyclometalated ruthenium(II) complexes have been successfully applied in homopolymerizations and copolymerizations of hydrophobic and hydrophilic monomers [21,24,25,26]. The use of cyclometalated ruthenium(II) complexes can eliminate the undesired copper–promoted side reactions. However, the radical polymerization of AMPS using ruthenium catalyst has not been evaluated yet. Therefore, in this study we present the ATRP synthesis of AMPS catalyzed by [Ru(*o*-C_6_H_4_-2-py)(phen)(MeCN)_2_]PF_6_ complex (phen: 1,10-phenanthroline), without previous protection of the acid groups. The copolymerization approach was carried out by sequential polymerization of a second monomer, methyl methacrylate (MMA) and 2-hydroxyethyl methacrylate (HEMA), respectively, via direct fresh feeding into the PAMPS prepolymer solution.

## 2. Materials and Methods

### 2.1. Materials and Reagents 

All reactions were carried out under inert atmosphere using conventional Schlenk techniques. 2-Acrylamido-2-methylpropane sulfonic acid (AMPS, 99%) and all reagents were supplied by Sigma-Aldrich, México. The 2-hydroxyethyl methacrylate (HEMA, 99%) and methyl methacrylate (MMA, 99%) monomers were purified by passing through an inhibitor remover column. Methanol (MeOH, 99.9%) was distilled prior to use. *N,N*-dimethylformamide (DMF, 99.8%), methanol and water were previously deoxygenated with nitrogen bubbling for 20 min before use. Ethyl 2–bromoisobutyrate (EBiB) and deuterated solvents were used as received.

### 2.2. Synthesis of the Ruthenium(II) Catalyst

**Figure 1:**
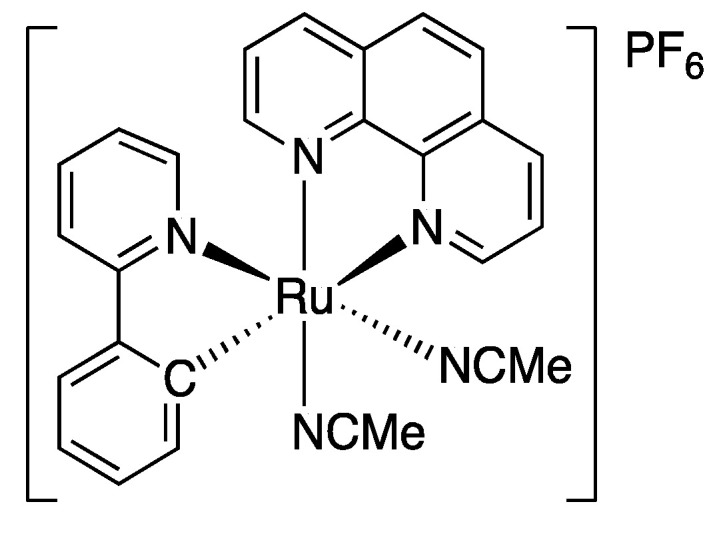
Chemical structure of the [Ru(*o*-C_6_H_4_-2-py)(phen)(MeCN)_2_]PF_6_ catalyst.

The [Ru(*o*-C_6_H_4_-2-py)(phen)(MeCN)_2_]PF_6_ catalyst was synthesized according to the literature [27,28]. The chemical structure of the ruthenium catalyst is presented in Figure 1.

### 2.3. Synthesis of the Homopolymers and Copolymers

Radical homopolymerization of AMPS. The reaction was carried out in solution with a 200:1:1 mol ratio between the monomer, initiator and the catalyst, respectively. In a typical PAMPS synthesis, a 25 mL Schlenk flask was charged with [Ru(*o*-C_6_H_4_-2-py)(phen)(MeCN)_2_]PF_6_ (25 mg, 0.037 mmol), AMPS (1.56 g, 7.52 mmol) and solvent (2 mL DMF). The homogeneous solution was degassed by three freeze–pump–thaw cycles and EBiB (5.5 μL, 0.037 mmol) was added. The reaction mixture was immersed in an oil bath with stirring at 80 °C for 10 h. Samples were taken after certain time intervals using a N_2_-purged syringe. The polymer was poured into ethyl acetate, filtered and finally purified through a Florisil column with methanol.

*Synthesis of PAMPS-b-PHEMA copolymer*. The PAMPS macroinitiator was first polymerized under the conditions described above using DMF as solvent at 80 °C. The reaction was stopped after 16 h and 0.6 mL of DMF was added to decrease the high viscosity of the reaction mixture. Then, 0.9 mL HEMA was added by syringe under N_2_ purge. After complete homogenization, the reaction was carried out at 70 °C for 3 h. The purification procedure was similar to that of PAMPS homopolymerization. Molar composition for the block copolymerizations was controlled by varying the HEMA ratio.

#### Synthesis of PAMPS-b-PMMA Copolymer

A solution of AMPS (0.39 g, 1.88 mmol), [Ru(*o*-C_6_H_4_-2-py)(phen)(MeCN)_2_]PF_6_ (12.5 mg, 0.0185 mmol) and EBiB (2.75 μL, 0.0185 mmol) in 0.5 mL DMF was heated at 80 °C for 16 h. Then 0.2 mL or 0.5 mL of degassed MMA was added by syringe under N_2_ purge for a copolymer with 75 and 88% mol of PMMA block, respectively. After complete homogenization, the flask was placed in an oil bath at 80 °C for 20 h. The reaction mixture was cooled and poured into diethyl ether and the precipitate was filtered. Finally, the polymer was dissolved in dichloromethane and purified by passing through a silica gel column to remove the residual catalyst.

### 2.4. Characterization and Membrane Preparation

The conversions were determined by ^1^H-NMR on a Varian NMR spectrometer (Agilent technologies, Santa Clara, CA, USA) operating at 600 MHz using deuterium oxide (D_2_O) as the deuterated solvent. The monomer conversion (C) was calculated as reported by [29].

(1) $$\;C=100\times \left(1-\frac{I_{A}}{I_{B}}\right)\;\mathrm{or}\;C=100\times \left(1-{\;I}_{A}\right)$$

where *C* is the monomer conversion, *I_A_* is the integrated signal between 6.13 to 6.28 ppm corresponding to CH_2_ protons adjacent to the double bond in the monomer, and *I_B_* is the integrated signal at 3.44 ppm due to CH_2_ protons adjacent to the –SO_3_H group in the monomer and polymer (this peak was selected as a reference *I_B_* = 1).

Molecular weight (*M_n_*) and dispersity (Ð) were determined by gel permeation chromatography (GPC) on an Agilent 1100 HPLC equipped with a refractive index detector (IR) and two columns: Zorbax PSM 60-S and PSM 1000-S (Agilent technologies, CA, USA). DMF containing LiBr (0.05%) was used as the eluent (flow rate 0.70 mL min^−1^) at 30 °C and PMMA was used as the calibration standard.

### 2.5. Membrane Preparation

Membranes were prepared by casting a 6% solution of PAMPS-*b*-PHEMA and PAMPS-*b*-PMMA copolymers in deionized water and methanol, respectively.

*PAMPS-b-PMMA membrane*. A total of 410 mg of PAMPS-*b*-PMMA was dissolved in 6.8 mL of methanol at room temperature for 5 h under continuous stirring until a homogeneous and highly viscous solution was obtained. The solution was poured into an aluminum ring and the solvent was evaporated slowly at 30 °C for 24 h. Finally, the obtained membranes were dried in a vacuum oven at 80 °C for 24 h to eliminate the residual solvent.

*PAMPS-b-PHEMA membrane*. A total of 410 mg of PAMPS-*b*-PMMA was dissolved in 6.8 mL of deionized water at 60 °C for 24 h under continuous stirring. The polymer solution was then cooled and sulfosuccinic acid (15 wt% SSA/weight of PHEMA block) was added. In the next step, the solution with SSA was stirred for 12 h and an additional 2 h in an ultrasonic bath. The PAMPS-*b*-PMMA solution was poured onto a Teflon plate and the solvent was evaporated slowly at 50 °C for 24 h. The obtained membranes were dried in a vacuum oven at 60 °C for 24 h. Finally, the membranes were crosslinked at 120 °C for 1 h.

### 2.6. Determination of Ion Exchange Capacity (IEC)

The ion exchange capacity (IEC, mmol/g) value of the membranes was calculated using an acid–base titration method. Membrane samples of 0.1 g were immersed in 5 mL of NaOH solution (0.1 M) at room temperature for 24 h to be converted into their sodium salt form. Subsequently, the remaining solution was titrated with 0.02 M HCl. The IEC value was calculated according to Equation (2):

(2) $$\mathrm{IEC}\;\left({\mathrm{mmol}\;g}^{-2}\right)=\frac{(V_{\mathrm{NaOH}})\left(C_{\mathrm{NaOH}}\right)-(V_{\mathrm{HCl}})\left(C_{\mathrm{HCl}}\right)}{W_{s}}$$

where V_NaOH_ and C_NaOH_ are the volume and molarity of NaOH, respectively; V_HCl_ and C_HCl_ are the volume and molarity of HCl consumed in the titration, respectively; and W_s_ is the dry weight of the samples. The IEC was determined in duplicate.

## 3. Results and Discussion

### 3.1. Synthesis and Characterization of AMPS Homopolymer

**Figure 2:**
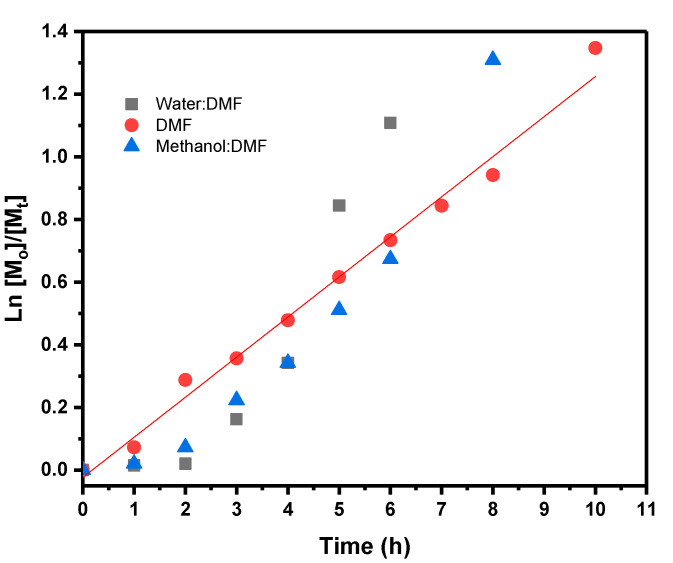
Kinetic plot of poly(2-acrylamido-2-methyl-1-propanesulfonic acid) (PAMPS) synthesized by [Ru(*o*-C_6_H_4_-2-py)(phen)(MeCN)_2_]PF_6_ in different solvents.

Initially, the polymerization of AMPS was carried out using a mixture of water:DMF or methanol:DMF (50:50, *v*/*v*), as reported in the literature (See Table 1) [14,15,30]. As shown in Figure 2, the study of the reaction kinetics in water:DMF showed a slow initiation, with an induction period of around 2 h and a considerable increase in viscosity from the fourth hour. This can be attributed to low efficiency of the initiator caused by the decrease of the solubility of EBiB in aqueous media or the spontaneous polymerization capacity of this type of acid monomer in aqueous solutions [21,31,32]. With regard to the spontaneous polymerization of AMPS in the presence of water, the dependence of the initial polymerization rate on the neutralization degree was reported. In absence of a neutralizing agent (NaOH), the AMPS polymerization proceeds slowly, followed by a considerable increase in the reaction viscosity and reaction rate [31]. Another possible explanation for the induction period could be the slow generation of the actual catalytically active system from the [Ru(*o*-C_6_H_4_-2-py)(phen)(MeCN)_2_]PF_6_ precatalyst, possibly involving the oxidation of Ru(II) to Ru(III), as in the generally accepted mechanism for ATRP [24]. Furthermore, polymers were obtained with high molecular weights (M_n_) and an increase of the Ð values was observed (> 1.7) (Table 1), though at low conversions. The polymerization of AMPS in methanol:DMF proceeded with a similar behavior to the reaction in water:DMF. 

On the other hand, the polymerization of AMPS in DMF catalyzed by [Ru(*o*-C_6_H_4_-2-py)(phen)(MeCN)_2_]PF_6_ was slightly slower—reaching a conversion of 61% in 8 h and 74% in 10 h—than the aforementioned systems. Nevertheless, it presented a pseudo-first-order kinetic (Figure 1), which indicate that the concentration of active species in propagation remains constant during the reaction [14,33,34] and the induction period disappeared, as shown in Figure 2. This behavior is characteristic of a quasiliving polymerization [33,34], which can be attributed to the dramatic increases of the solubility of both the catalyst and EBiB initiator in DMF that leads to a higher efficiency of the initiator and fast generation of the catalytically active species.

**Figure 3:**
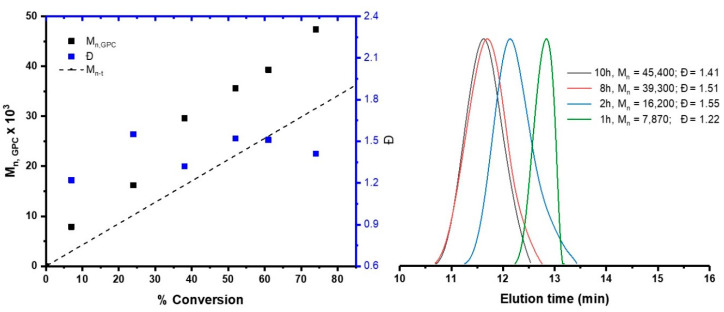
Evolution of the molecular weight (M_n_) and dispersity (Ð) with the conversion for AMPS polymerization in DMF catalyzed by [Ru(*o*-C_6_H_4_-2-py)(phen)(MeCN)_2_]PF_6_ at 80 °C. [AMPS]_o_:[EBiB]_o_:[catalyst]_o_ = 200:1:1.

As can be seen in Figure 3, the M_n_ increased with conversion and a narrow molecular weight distribution is obtained (in the range of 1.22–1.55). A discrepancy was observed between the theoretical (M_n-t_) and experimental M_n_ values due to the difference in the hydrodynamic volume between PAMPS, which is hydrophilic, and the hydrophobic calibration standard (PMMA) [21,33,35].

### 3.2. Synthesis of Block Copolymers 

PAMPS is a water-swollen homopolymer to the point of being soluble. Thus, to be used for potential applications, such as polyelectrolyte membrane fuel cells, a catalytic membrane for biodiesel production or for medical applications, it can only be used copolymerized, crosslinked, blended or in gel forms to control its swelling in aqueous solution [4,36]. In this work, PAMPS-*b*-PHEMA and PAMPS-*b*-PMMA copolymers were synthesized in order to prove the effectiveness of [Ru(*o*-C_6_H_4_-2-py)(phen)(MeCN)_2_]PF_6_ catalyst in a living polymerization using PAMPS as macroinitiator.

#### 3.2.1. Synthesis of PAMPS-*b*-PMMA Block Copolymer

Amphiphilic block copolymers are very interesting due to their applications, properties and morphological self-assembly capacity. However, the synthesis of amphiphilic block copolymers is not an easy task, first due to the incompatibility of each segment of different nature (hydrophilic and hydrophobic) and second due to the difficulty of finding a common solvent for both blocks. The synthesis is even more complicated when the hydrophilic block possesses acidic functional groups [37]. Considering that the most active monomers (methacrylates) are polymerized before the least active ones (acrylamides) in the copolymers synthesis [38], copolymerization was carried out using previously isolated PMMA (synthesized under similar conditions described for the AMPS homopolymerization in THF at 80 °C for 4 h) as macroinitiator in DMF at 80 °C. Nevertheless, the copolymerization did not proceed under these conditions. However, the PAMPS-*b*-PMMA copolymer was successfully obtained with the one-pot sequential method with the addition of MMA monomer to the PAMPS macroinitiator. This can be attributed to the presence of the polar groups (–SO_3_H) of AMPS which may be acting as accelerators in the system and therefore increasing the reactivity of the AMPS monomer compared to MMA [39,40,41]. The effect of acceleration on the ATRP by polar groups (either from polar additives, solvents or monomers containing polar substituents) when using Cu catalyst has been attributed to the increase in polarity in the reaction medium and the generation of more active catalytic species through the interaction of polar molecules with the metallic center [39,40,41]. However, so far, we do not have evidence of some kind of AMPS interaction with the ruthenium catalyst. We will be carrying out in-depth future research on this specific topic. On the other hand, Oikonomou et al. reported the synthesis of a series of amphiphilic copolymers of poly(sodium styrene sulfonate)-*b*-poly(methyl methacrylate) (PSSNa-*b*-PMMA) by ATRP using PSSNa macroinitiator [37], in a similar way to the synthesis of copolymers reported in this work.

The PAMPS macroinitiator was synthesized previously for 16 h to ensure high monomer consumption (conversion of 82%). It is worth noting that a 100:1:1 molar ratio was used for the monomer/catalyst/initiator to obtain a low molecular weight macroinitiator that would facilitate growth of the second polymer block. With this method, PAMPS-*b*-PMMA block copolymers with 10 and 25% mol PAMPS were synthesized. In contrast to the AMPS homopolymer, the PAMPS-*b*-PMMA copolymers were insoluble in water and soluble in dichloromethane.

**Figure 4:**
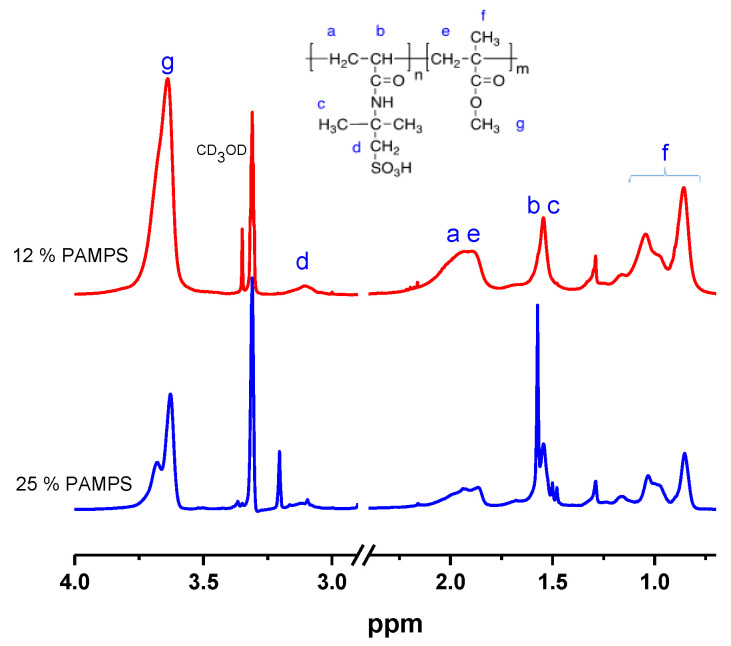
^1^H-NMR (CD_3_OD) of poly(2-acryloamido-2-methylpropanesulfonic acid)-*b*-poly(methyl methacrylate) (PAMPS-*b*-PMMA) with 12 and 25% mol of PAMPS.

Detailed structural information for the PAMPS-*b*-PMMA synthesized was obtained by ^1^H-NMR. As can be seen in Figure 4, the PAMPS-*b*-PMMA spectra exhibit signals from both PAMPS and PMMA polymeric blocks. The PAMPS signals at 3.22, 1.92, 1.58 and 1.37 ppm correspond to methylene protons (*d*) adjacent to the acid group, polymer backbone protons (*a*, *b*) and methyl protons (*c*), respectively [29,42]. The assignments of PMMA are shown directly in the spectra in accordance with the literature [24]. The composition of the copolymers was obtained by ^1^H-NMR from the integration of the PAMPS signal at 3.08 ppm (signal *d*) and at 3.65 ppm (signal *g*) for PMMA and the factors 2 and 3 derive from the involved protons in each signal, according to the relationship:

(3) $${\mathrm{Molar}\;\mathrm{composition}}_{\mathrm{PAMPS}}=\frac{3\left(I_{3.08\;\mathrm{ppm}}\right)\;}{3\left(I_{3.08\;\mathrm{ppm}}\right)+2(I_{3.65\;\mathrm{ppm}})}$$

In Figure 4, we present the spectrum of two copolymers with an estimated block composition for the PAMPS/PMMA diblock of 12/88 and 25/75 (% mol).

**Figure 5:**
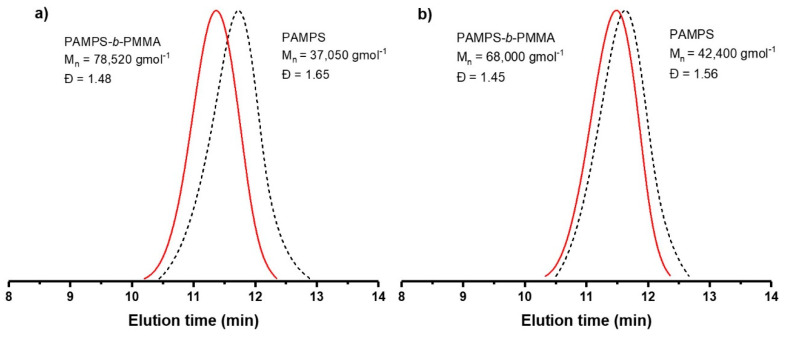
GPC elugram of PAMPS-*b*-PMMA copolymers with (**a**) 12% mol and (**b**) 25% mol of AMPS.

The GPC elugrams (Figure 5) confirmed the diblock structure of the PAMPS-*b*-PMMA copolymers. A significant increase is observed in molecular weight in copolymers with 12 and 25% mol PAMPS from 37.050 to 78.520 and from 42.400 to 68.000, respectively, with a reduction in the Ð with respect to the PAMPS macroinitiator, indicating the efficient formation of the copolymer. Furthermore, the curves of the copolymers were monodispersed and symmetrical, attributed to the absence of impurities or dead chains of the macroinitiator [21,43]. It is worth noting that the copolymers presented bimodal curves when they originated from a PAMPS macroinitiator synthesized in a reaction of less than 16 h, due to the propagation of the MMA monomer with unreacted AMPS monomer [11]. It is important to mention that the one-pot sequential method has the disadvantage that the propagation of the second monomers could involve a mixture of the second monomer with unreacted first monomer, resulting in a second block as a random copolymer [11]. In this case, such “contamination” of the second block in the PAMPS-*b*-PMMA copolymerization would be difficult to identify. However, we did not observe a tailing of the molecular mass distribution towards lower molar masses or bimodal curves in GPC chromatogram (see Figure 5). Moreover, we identified the characteristic signals of the two PAMPS and PHEMA blocks in RMN spectra that cannot be identified in random PAMPS-*co*-PMMA, in particular the signal at 3.08 ppm (signal *d* in Figure 4) corresponding to the protons of methylene closest to the sulfonic acid group [1,10].

#### 3.2.2. Synthesis of PAMPS-*b*-PHEMA Block Copolymer

Copolymers with double hydrophilic blocks are also highly attractive, because most of them are biocompatible and present stimuli-responsive and adjustable amphiphilic properties with self-assembly capacity in solution [44,45]. Poly(2-acryloamido-2-methylpropanesulfonic acid)-*b*-poly(2-hydroxyethylmethacrylate) (PAMPS-*b*-PHEMA) block copolymers were successfully synthesized in the system catalyzed by [Ru(*o*-C_6_H_4_-2-py)(phen)(MeCN)_2_]PF_6_ using PAMPS as macroinitiator. PAMPS-*b*-PHEMA copolymers were synthesized with molar compositions of PHEMA between 28 and 36% mol. In polymerization reactions with higher HEMA concentrations, obtained copolymers were insoluble in the solvents, such as DMF, DMSO and methanol, even upon heating. This suggests that some degree of crosslinking occurred during the polymerization, possibly due to intermolecular interactions and the formation of hydrogen bonds between the different functional groups present in the two blocks. In addition, it was necessary to reduce the reaction temperature to 70 °C on adding the second charge of monomer to minimize the formation of insoluble crosslinked products.

**Figure 6:**
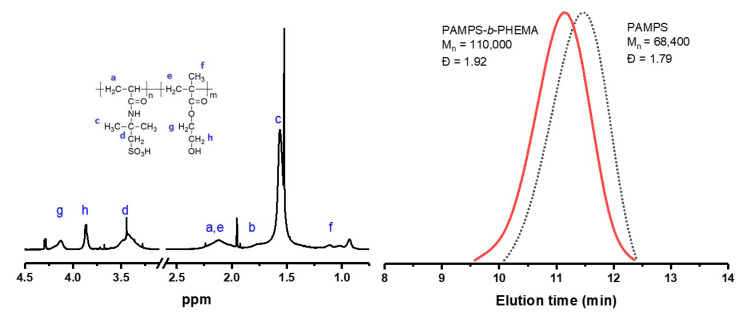
^1^H-NMR (D_2_O) y GPC elugram of poly(2-acryloamido-2-methylpropanesulfonic acid)-*b*-poly(2-hydroxyethylmethacrylate) (PAMPS-*b*-PHEMA) catalyzed by [Ru(*o*-C_6_H_4_-2-py)(phen)(MeCN)_2_]PF_6_.

Figure 6 shows the ^1^H-NMR spectrum and the GPC curves of the copolymer synthesized with a molar composition percentage of 65/35 (% mol, PAMPS/PHEMA). The ^1^H-NMR analysis confirmed the chemical structure of the copolymer and the characteristic signals of the two PAMPS and PHEMA blocks were identified, matching those reported in the literature [21,46]. The molar composition of the copolymers was calculated by integration of the peaks at 3.43 and 3.86 ppm associated with the methylene protons adjacent to the –SO_3_H group of PAMPS and the –OH group of PHEMA, respectively. The GPC analysis also confirmed the diblock structure of the PAMPS-*b*-PHEMA copolymer. The molecular weight of the copolymer increased when compared to the PAMPS macroinitiator and the GPC curve of the copolymer was broader (Ð = 1.92), this was due the percentage of dead chains in the PAMPS macroinitiator as result of high molecular weight and high percentage conversion of the first block [11].

In general, the copolymerization of PAMPS-*b*-PMMA and PAMPS-*b*-PHEMA was carried out in a quasiliving polymerization without the protection and deprotection of the AMPS monomer, which demonstrated the efficiency of the cyclometalated [Ru(*o*-C_6_H_4_-2-py)(phen)(MeCN)_2_]PF_6_ complex and confirmed the living character of this polymerization system in DMF.

### 3.3. Ion Exchange Capacity (IEC) of Membranes

The PAMPS-*b*-PHEMA and PAMPS-*b*-PMMA copolymers showed excellent solubility in various solvents, which allowed obtaining flexible and transparent membranes. These membranes were used to calculate the ion exchange capacity (IEC, mmol H^+^/g) or the amount of the ion-exchangeable [H^+^] sulfonic groups, and the results are presented in Table 2.

The IEC values of the PAMPS-*b*-PMMA membranes ranged from 0.55 to 1.40 mmol H^+^/g, very close to the theoretical values. The PAMPS-*b*-PHEMA membrane was previously crosslinked with sulfosuccinic acid (SSA) at 120 °C for 1 h to prevent it from dissolving in aqueous NaOH solution. The experimental IEC value of PAMPS-*b*-PHEMA was slightly higher (3.35 mmol H^+^/g) than the theoretical value (3.10 mmol H^+^/g), due to the contribution of –SO_3_H groups of the SSA crosslinking agent. Thus, the crosslinking reaction in PAMPS-*b*-PHEMA membrane has a dual function: 1) To make the membrane more stable, and 2) to increase the IEC or the number of acid sites of the materials. The IEC values of these membranes depend strongly on the molar composition of PAMPS in the diblock copolymer and the balance between hydrophilic-hydrophobic behavior from the comonomer used (MMA or HEMA).

## 4. Conclusions

The polymerization of AMPS without previous protection of its strong acid groups catalyzed by [Ru(*o*-C_6_H_4_-2-py)(phen)(MeCN)_2_]PF_6_ in DMF at 80 °C was investigated. The radical polymerization was carried out successfully and its living character was confirmed by the syntheses of PAMPS-*b*-PMMA and PAMPS-*b*-PHEMA. The well-defined copolymers were obtained by one-pot sequential method, with the addition of the second monomer (MMA or HEMA) into a PAMPS macroinitiator. The combination of PAMPS macroinitiator with MMA and HEMA comonomer produced an amphiphilic block copolymer and a double hydrophilic block copolymer, respectively. A higher molar composition of PAMPS increases the ionic exchange capacity of the membranes, which can be attributed to the greater number of sulfonic acid groups present. Given the well-defined structure, IEC and excellent membrane-forming ability of block copolymers, these materials could be adequate for use in polyelectrolyte membrane fuel cells or as catalytic membranes for biodiesel production. We are working on these main applications and the results will be reported in a forthcoming publication, where, in particular, the relationship between the morphology of the block copolymer membranes with diffusion and permeability properties will be discussed.

## Figures and Tables

**Table 1:** Polymerization of 2-acrylamido-2-methyl-1-propanesulfonic acid (AMPS) mediated by [Ru(*o*-C_6_H_4_-2-py)(phen)(MeCN)_2_]PF_6_ in different solvents. [AMPS]_o_:[EBiB]_o_:[catalyst]_o_ = 200:1:1.

Solvent	Time (h)	Conversion (%)	M_n_ × 10^3^ g/mol	Ð
Water:DMF	6	68	88.3	1.83
Methanol:DMF	8	73	75.2	1.75
DMF	8	61	39.3	1.51

**Table 2:** Ion exchange capacity (IEC) of PAMPS-*b*-PMMA and crosslinked PAMPS-*b*-PHEMA membranes.

Copolymer	PAMPS %mol	Theoretical IEC (mmol H^+^g^–1^)	Experimental IEC (mmol H^+^g^–1^)
PAMPS-*b*-PMMA	12	0.58	0.55
	25	1.21	1.40
PAMPS-*b*-PHEMA	65	3.13	3.35
